# Supplementary material for: The interactome of intact mitochondria by cross-linking mass spectrometry provides evidence for coexisting respiratory supercomplexes
Source: Mol Cell Proteomics. 2017 Dec 8;17(2):216–32. doi: 10.1074/mcp.RA117.000470 (PMC5795388; doi:10.1074/mcp.RA117.000470)
Supplement: Supplemental Data [file supp_RA117.000470_133922_0_supp_23978_fzffwf.zip › spectra_annotation/mito_spectra_annotation/213-1-49-1-13-1.pdf]

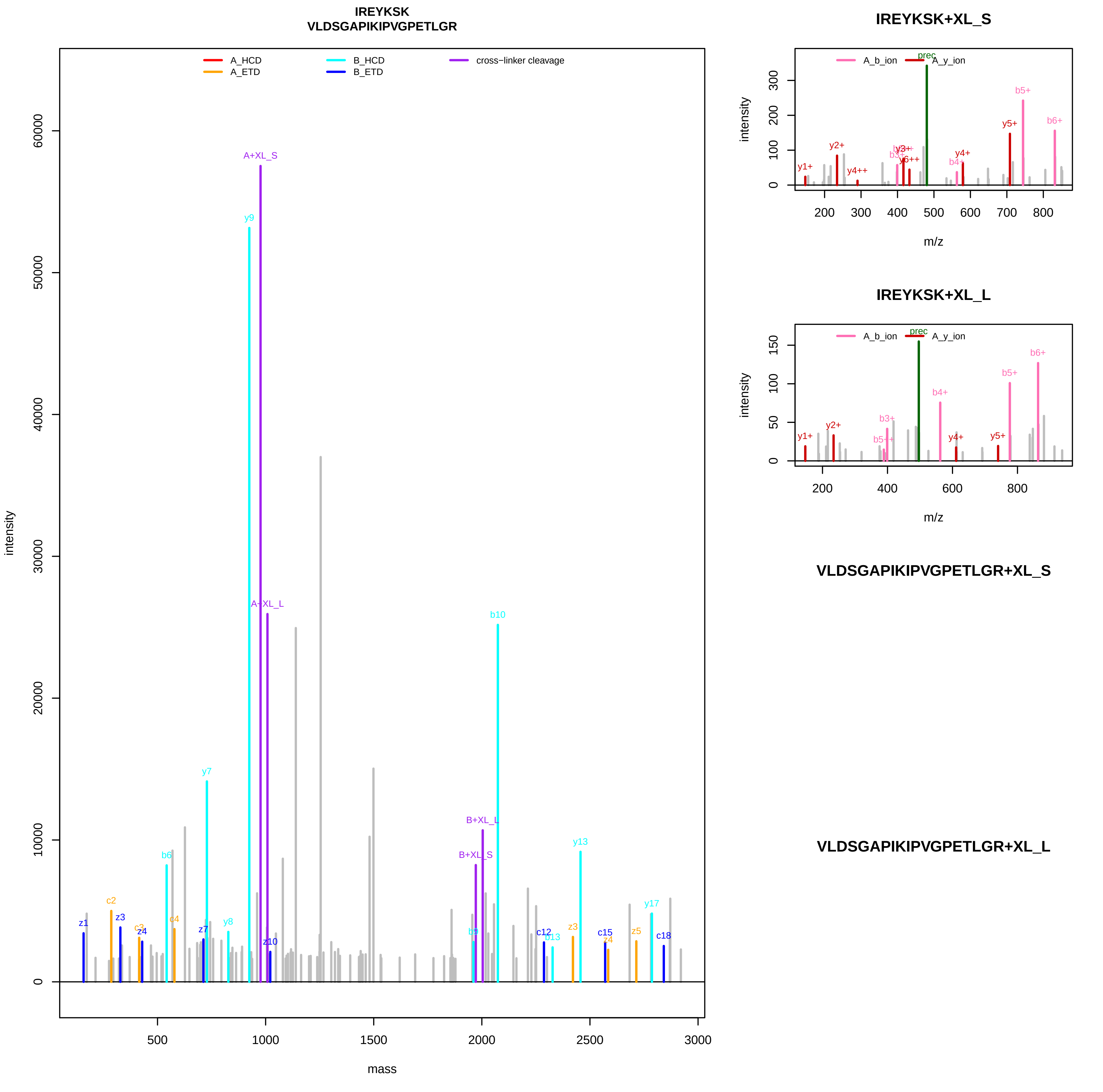
IREYKSK
VLDSGAPIKIPVGPETLGR
A_HCD
A_ETD
B_HCD
B_ETD
cross−linker cleavage
A+XL_S
A+XL_L
B+XL_L
B+XL_S
y9
y7
y8
y13
y17
b6
b9
b10
b13
c2
c3
c4
c12
c15
c18
z1
z3
z4
z5
z7
z10
intensity
mass
IREYKSK+XL_S
IREYKSK+XL_L
A_b_ion
A_y_ion
prec
y1+
y2+
y3+
y4+
y5+
y4++
y6++
b3+
b4+
b5+
b6+
b5++
m/z
VLDSGAPIKIPVGPETLGR+XL_S
VLDSGAPIKIPVGPETLGR+XL_L
